# Necrology

**Published:** 1901-03

**Authors:** 


### NECROLOGY.

It is our sad duty to report the death of three men in our ranks who have been distinguished leaders in veterinary medicine—honorable and brilliant professional men and personally dear and lovable friends and companions to all who knew them. The two Americans, Josiah H. Stickney, of Boston, and Albert W. Clement, of Baltimore, were pioneers in the development of veterinary medicine in this country. William Williams, a Welshman by birth, a Scotchman by adoption, and a cosmopolitan in the esteem and affection of the world, was a leader in the refinement of veterinary medicine in Great Britain.

### Josiah H. Stickney.

Josiah H. Stickney was one of the first veterinarians in the United States to bring veterinary medicine before the public as a scientific and honorable profession, and in his courteous manners, scientific knowledge, and professional abilities he furnished a constant example of an ideal professional life.

Josiah Henry Stickney was born in Boston, February 11, 1826. His father, Josiah Stickney, of Watertown, was a merchant and banker, president of the Market Bank and of the Massachusetts Horticultural Society, and a director of the Western Railroad and also of its successor, the Boston and Albany Railroad.

After attending the well-known private school of Gideon F. Thayer, in Chauncy Place, Mr. Stickney, at his father's desire, entered business in 1849 as a member of the firm of Cutler & Stickney, at 1 India Street. The paint, oil, and glass trade was not congenial to him and in 1854 he commenced to study medicine with Dr. Daniel D. Slade; later he attended the Tremont and Harvard Medical Schools. While a student he spent one year as house-surgeon at the Massachusetts General Hospital. He graduated in 1858 with the degree of M.D.

Dr. Stickney then went as surgeon on one of Train's vessels, sailing to England. On his arrival in that country he entered the Royal Veterinary College at London, from which he obtained his M.R.C.V.S., April 30, 1859, with high honors. When he returned in 1860 a severe cattle plague of pleuropneumonia was raging in Massachusetts. Governor Banks appointed the young physician as a member of the State Board of Health. His professional and executive ability was the power behind Dr. E. F. Thayer and the Cattle Commission in the wonderful eradication of contagious pleuropneumonia from Massachusetts. He continued in active veterinary practice until within a few years.

Dr. Stickney was the first President of the United States Veterinary Medical Association (now the American Veterinary Medical Association), in 1863–64. He took an active part in the Association for many years, and in later years, when he was personally prevented from attending, his keen interest was unabated. His judgment and advice in public matters were always good, and not biased by prejudice or personal influence.

His honesty and integrity in business matters was an unrivalled example which stamped its character on the younger men who surrounded him. Personally he was an entertaining and delightful companion.

Dr. Stickney was married in Waltham, Mass., October 8, 1861, to Elizabeth Shannon, daughter of Jonas C. March, of Boston. His widow and four children—Mary W., wife of Henry K. Swinscoe, of Philadelphia; Ellen F. Stickney, Josiah Stickney, of Clinton, and I. Lombard Stickney, of Jamaica Plain—survive him.

Dr. Stickney died on February 5, 1901, at the Massachusetts General Hospital, where he had served as a house-surgeon forty-three years before.

### Albert W. Clement.

Albert W. Clement, a recent President of the American Veterinary Medical Association (1898–99), belonged to the present generation, and his premature death comes as a sad blow to the profession and to his friends.

Albert W. Clement was born in Lawrence, Mass., in 1857, where he received his early education in the public schools. He then spent two years at Harvard College, Cambridge, Mass., where he took a special preliminary medical course. His health had been somewhat impaired at this time by an attack of rheumatism and a severe bone injury, and upon the advice of his physician he devoted himself to out-door occupation with horses, which led him to his desire to study veterinary medicine. This met with serious objection from his father on account of the estimation in which the profession was then held, but he was finally allowed to make a trial, with the warning that he would find one year enough.

In 1879 he went to McGill University, at Montreal, Canada, where he graduated from the veterinary department in 1882. He remained there the following three years as a teacher. He was also employed during that time by the Canadian Government in investigating contagious diseases in animals and in the inspection of export cattle. In 1885 he went to Europe and remained for two years, studying at the London and Berlin Veterinary Schools, the Koch and Virchow laboratories at Berlin, and the Pasteur laboratory at Paris. He returned to Montreal in 1887 and shortly afterward went to Baltimore. For six years he pursued investigations in pleuropneumonia and scientific research, conducted at the Johns Hopkins Hospital laboratory.

He was also connected with the United States Bureau of Animal Industry and filled the office of State Veterinarian of Maryland for several years in a most efficient manner, instituting and developing many important reforms.

From the commencement of his practice of veterinary medicine in Baltimore, Dr. Clement became one of the leaders in organizing and raising the standard of the profession. He was a constant attendant at the meetings of the United States (later American) Veterinary Medical Association, in which he constantly served actively as an officer or member of the important committees. For a number of years he was an active and ardent member of the Committee on Army Legislation in the endeavor to secure from Congress a veterinary service in the United States Army.

In Maryland he took hold of public matters with his whole heart and secured the passage of a most efficient State law regulating veterinary practice and contagious diseases and requiring registration of veterinarians. He perfected this by following it up and seeing that the regulations were enforced.

Dr. Clement served as veterinarian at a number of horseshows, notably at that of the Open-air Horseshow in New York, and exhibited a decision and example which should be followed at all shows.

In private practice his professional skill, integrity, and honesty were, like those of Dr. Stickney, an example of practical application of what he urged at all times that veterinary practice should be.

Conservative at most times, Dr. Clement had the courage of his convictions and could be an ultraradical when necessity demanded.

Personally he had a constitution fitted to his fine physique, and no day or night was too long for work to be done or entertaining hospitality for his friends.

In addition to the medical and veterinary associations he was a member of the Maryland Club, the flag of which was at half-mast in his memory; Elk Ridge Fox Hunting Club, and the Pimlico Driving Club.

Dr. Clement died on March 3d, in the Johns Hopkins Hospital at Baltimore, after an illness of a month, from valvular disease of the heart. Funeral services were held at his residence on March 5th, and the interment took place at Lawrence, Mass., on March 7th.

### William Williams.

The late Principal Williams of the New Veterinary College, Edinburgh, was best known to most of the veterinarians in America as the author of his text-books, which have served in all colleges—that on *Veterinary Surgery*, which has passed through nine editions, and that on *Veterinary Medicine*, which is now in the eighth edition—but he leaves many who knew him personally, with happy recollections of his delightful personality and esteem for his distinguished labors.

Principal Williams was born at Bontnewydd, Wales, in 1832. He was descended from a race of veterinary surgeons who, prior to the college qualifications, were known as farriers, and he was the fifth of the family in direct line who had made their livelihood mainly as doctors of horses and cattle. At the age of twenty years, after having been a student for three years with a Lancashire veterinary surgeon, his health failed him and he went to Australia, where he had a varied experience as miner, surveyor, etc.

He returned in 1855 and entered the Dick Veterinary College.

He won the Highland Society's first gold medal. After practising in Yorkshire he was, upon the death of Professor Dick, appointed principal of the Dick College in 1886, and devoted himself to this work until 1872. In 1872 he became a member of the Council of the Royal College of Veterinary Surgeons, and in 1879–80 was President of that body. Professor Williams was a Fellow of the Royal Society of Edinburgh and a Justice of the Peace for that city. In 1872 he founded the college known as the New Veterinary College in Edinburgh. During the last eight years he has been a member of the Town Council of Edinburgh.

Professor Williams on several occasions took an active part in the defence of cattle imported from America, which the English government was endeavoring to prove to have contagious pleuro-pneumonia, but which did not.

He was sent to Jamaica to investigate an outbreak of cattle disease there, and was consulted by the government in regard to the establishment of the "Indian Civil Veterinary Department."

As a teacher Principal Williams was a strict disciplinarian, and the prizes won by his students sustained his methodical teaching.

As a man he was genial and humorous, with a broad strain of Celtic wit.

### Thomas Hagyard.

Kentucky has just lost by death one of her prominent veterinarians, Dr. Thomas Hagyard, of Lexington. Dr. Hagyard died of rheumatism of the heart, at Elmendorf Stock Farm.

Dr. Hagyard was well known and a great favorite with his colleagues and with horsemen. He was born at Brampton, Ontario, Canada, forty-seven years ago, and was educated and graduated as a veterinary surgeon at the Ontario College at Toronto. In 1878 he removed to Kentucky with his family, when the shorthorn business was on a boom, and located at Winchester, where he immediately acquired a large practice. In 1881 he removed to Nashville, Tenn., where he remained until about two years ago, when he engaged with J. B. Haggin to look after the health of the horses at Elmendorf. While in Tennessee he was surgeon to the Hermitage Stud, when Wedgewood and Bow Bells were there; to Belle Meade Farm, the famous stud of General Jackson, and to the home of St. Blaize, owned by Charles Reade, and was finally engaged exclusively to Mr. Haggin, with whom he remained until his death. He was born of a family of veterinary surgeons. His father, Dr. E. T. Hagyard, was a Scottish surgeon and graduated at Edinburgh, and his two brothers, Dr. E. W. Hagyard, now at Marcus Daly's Bitter Root Farm, Hamilton, Montana, and Dr. John R. Hagyard, of Lexington, survive him. The deceased leaves a widow and three sisters. His remains were buried in the Lexington Cemetery.

